# Interpopulation Similarity of Sex and Age-Related Body Composition Variations Among Older Adults

**DOI:** 10.3390/ijerph17176047

**Published:** 2020-08-20

**Authors:** Elisabetta Marini, Roberto Buffa, Luis Alberto Gobbo, Guillermo Salinas-Escudero, Silvia Stagi, Carmen García-Peña, Sergio Sánchez-García, María Fernanda Carrillo-Vega

**Affiliations:** 1 Department of Life and Environmental Science, Neuroscience and Anthropological Section, University of Cagliari, Cittadella Universitaria, Monserrato, 09042 Cagliari, Italy; emarini@unica.it (E.M.); rbuffa@unica.it (R.B.); silviastagi89@gmail.com (S.S.); 2 Department of Physical Education, School of Technology and Sciences, São Paulo State University (UNESP), Presidente Prudente, São Paulo 19060-900, Brazil; luis.gobbo@unesp.br; 3 Center for Economic and Social Studies in Health, Hospital Infantil de México Federico Gómez, Mexico City 06720, Mexico; guillermosalinas@yahoo.com; 4 Research Department, Instituto Nacional de Geriatría, Mexico City 10200, Mexico; mgarciapena@gmail.com; 5 Epidemiology and Health Services Research Unit, Aging Area, IMSS, Mexico City 06720, Mexico; ssanchezga71@gmail.com; 6 Geriatric Epidemiology Unit, Research Department, National Institute of Geriatrics, Instituto Nacional de Geriatría, Mexico City 10200, Mexico

**Keywords:** body composition, anthropometry, specific BIVA, elderly

## Abstract

The aim of the study was to analyze sex and age-related body composition variations among older adults from the Brazilian, Italian, and Mexican population. A cross-sectional analysis was conducted in 1103 community-dwelling older adults (634 women and 469 men), aged 60 to 89 years, living in Brazil (*n* = 176), Italy (*n* = 554), and Mexico (*n* = 373). Anthropometric measurements were taken, BMI was calculated, and impedance measurements were obtained (resistance, R, reactance, Xc). Specific bioelectrical impedance vector analysis (specific BIVA) was applied, with the specific vector defined by impedance, or vector length (Z = (Rsp^2^ + Xcsp^2^)^0.5^), and phase angle (PA = arctan Xc/R 180/π). Population, sex, and age differences in anthropometric and bioelectrical variables were evaluated by means of a two way ANOVA. The mean bioelectrical vectors were graphed by confidence ellipses and statistically compared by the Hotelling’s T^2^ test. The three population groups showed differences in body mass and composition (*p* < 0.001): the Brazilian sample was characterized by greater body dimensions, longer vectors (higher relative content of fat mass), and lower phase angles (lower skeletal muscle mass). Men were taller and heavier than women (*p* < 0.001) but had a similar BMI (*p* = 0.102). They also had higher phase angle (higher skeletal muscle mass) (*p* < 0.001) and lower vector length (lower %FM) (*p* < 0.001). In the three population groups, the oldest individuals showed lower anthropometric and phase angle values with respect to the youngest ones (*p* < 0.001), whereas the vector length did not change significantly with age (*p* = 0.665). Despite the differences between sexes and among populations, the trend of age-related variations was similar in the Brazilian, Italian, and Mexican older adults.

## 1. Introduction

Aging research is a fundamental tool for health systems, especially in countries where the growth rate of older adults is high. In Latin America and the Caribbean, older adults represent approximately 8% of the regional population [1]. In Mexico, just over 10% of the total population is older adults [2], and in Brazil the figure increases to 13% [3]. A different demographic panorama can be seen in the European Union, where about 27% of the total population is older adults [4]. Italy, with over 28% of older adults, is one of the countries with the most significant number of individuals in this group [5].

Regardless of the geographical area in which older adults live, and the demographic panorama of each place, the general trend of individual aging involves body composition variations, specifically on fluid distribution, fat, and muscle mass components. These variations expose the elderly to the risk of sarcopenia and sarcopenic obesity [6].

Although the age-related trend of body composition variations has been studied in various populations [7,8], to the best of our knowledge, few comparative analyses in a transnational perspective have been performed [9,10].

Practical methods such as bioelectrical impedance analysis (BIA), can be applied to reduce the procedural and cost limitations that arise from complex experimental designs, such as the extensive sampling of elderly people in an international context. However, the traditional BIA approach hides the risk of evaluation biases when used to compare different samples. Body composition predictive equations can be inadequate for all samples, and the use of population-specific equations can reduce the comparability of results. In this respect, specific bioelectrical impedance vector analysis (specific BIVA) [11], a ready to use and accurate technique for the semi-quantitative evaluation of body composition can be a valid alternative. This technique does not require predictive equations for the estimation of body compartments, but analyses raw bioelectrical data (resistance and reactance), standardized for body size (body length and cross-sectional areas), to neutralize the effect of anthropometric differences. The resulting specific resistance (Rsp, ohm cm) and reactance (Xcsp, ohm cm) can be projected on the Cartesian plane as vectors, defined by the impedance module (Z = (Rsp^2^ + Xcsp^2^)^0.5^) and the phase angle (arctan Xc/R 180/π). Specific BIVA has been compared with gold standard techniques, showing a positive correlation between vector length and body fat percentage (%FM) [11,12,13], and between phase angle and intracellular/extracellular water ratio (ICW/ECW) [13] and skeletal muscle mass [11]. The technique has been already applied in various research on older adults, in both physiological [12,14,15] and clinical conditions [16,17]. Hence, it seems particularly appropriate to be used for analyzing age-related body composition variations in older adults from different geographical areas.

## 2. Materials and Methods

### 2.1. Design and Settings

A cross-sectional analysis of three national studies was conducted in 1103 community-dwelling older adults (634 women and 469 men), aged 60 to 89 years, living in three different countries: Brazil (176 individuals), Italy (554 individuals), and Mexico (373 individuals) (Table 1).

The Brazilian multicentric study refers to the last wave of the cohort study “Influence of physical activity on sarcopenia, sarcopenic obesity, ‘dysmobility’ syndrome and functional disability in the elderly”: a 24-month cohort, conducted between January 2015 and May 2017 in the city of Presidente Prudente (Southeastern Brazil) [18]. In a first moment, individuals ≥ 60 years were recruited in two Public Health Services selected by the Municipal Health Secretary of the city. In a second phase, the invitation was extended to individuals in the same age group in the general population. The sample selection was carried out by convenience sampling. This study was approved by the Ethical Committee at the São Paulo State University (UNESP)/Presidente Prudente (approval code: CAAE 26058114.3.0000.5402).

The Italian study group is part of the project “Specific bioelectrical impedance vector reference values for healthy older adults Italian population (65–100 years)” [14]. The sample selection was carried out by random sampling. For the present analysis, individuals aged more than 90 years were excluded. The Ethical Committee of Cagliari University Hospital approved the study (approval code: PG/2014/21461).

The Mexican study refers to the third wave of the project “Cohort of obesity, sarcopenia and frailty of Older Mexican Adults”, conducted between April 2014 and July 2016 [19]. The study included adults ≥ 60 years of age selected through simple random selection from the list of older people affiliated with the 41 Family Medicine Units of the Mexican Institute of Social Security from Mexico City. The study was approved by the National Commission of Scientific Research of the Mexican Institute of Social Security (IMSS, approval code: 2012-785-067).

In all countries, all the individuals were recruited after informed consent. Exclusion criteria for the present analysis included persons not living in a long-stay institution or using implanted electrical devices, as well as diagnosis of pulmonary disease, severe cardiovascular or uncontrolled metabolic diseases, cancer, inflammatory conditions, human immunodeficiency virus/acquired immunodeficiency syndrome, tuberculosis, and chronic kidney disease.

### 2.2. Measurements

The surveys were performed by trained personal in the three countries. All measurements were made in the morning. Participants were invited to avoid eating or drinking at least 4 h before the examination. Anthropometric measurements (weight; height; waist, calf, and arm circumferences) were taken in agreement with international criteria [20]. BMI was calculated as the ratio of body mass to height squared (kg/m^2^). Impedance measurements (resistance, R, reactance, Xc) were obtained using a single-frequency analyzer: the BIA 101 (Akern, Florence, Italy) device, with an operating frequency of 50 kHz at 800 μA, was used in Italy and Mexico, and the BIA Analyzer (Nutritional Solutions, Harrisville, MI, USA), with a frequency of 50 kHz at 450 μA, was used in Brazil. According to the standard procedure, whole-body BIA measurements were taken with the subject in a supine position and a leg opening of 45°. After cleaning the skin with alcohol, four electrodes were placed on the right hand and the right foot.

### 2.3. Statistical Analysis

Bioelectrical values obtained with different devices were made comparable by applying a correction factor calculated in a study showing that the Nutritional Solutions device underestimates Xc by 4.11 ohms and overestimates R by 3.98 ohms, with respect to Akern BIA101 [21]

Specific bioelectrical impedance vector analysis was applied [11, 12]. Specific resistance and reactance (resistivity, Rsp, ohm-cm, and reactivity, Xcsp, ohm cm) were obtained by multiplying R and Xc by a correction factor (A/L), where A is the estimated cross-sectional area (or 0.45 arm area + 0.10 waist area + 0.45 calf area, cm^2^) and L the length of the “conductor” (1.1 height, cm) of each individual. Impedivity (Zsp, ohm-cm) was calculated as (Rsp^2^ + Xcsp^2^)^0.5^ and phase angle (PA, degrees) as arctan Xc/R 180/π. Specific vectors were analyzed by means of their projection on the Cartesian plane defined by Rsp and Xcsp, where increasing values of impedivity, mainly due to resistivity, refer to higher values of the relative quantity of fat mass (%FM) and increasing values of phase angle to higher values of skeletal muscle mass and of intracellular/extracellular water ratio (ICW/ECW).

Population, sex, and age differences in anthropometric and bioelectrical variables were evaluated using a two way ANOVA. The mean bioelectrical vectors were also graphically represented by confidence ellipses and statistically compared employing Hotelling’s T^2^ test.

The level of significance was set at *p* < 0.05. Analyses were performed with the statistical package software SPSS Statistics, version x.18 (SPSS Inc., Chicago, IL, USA), the R Studio with the MASS library [22], and the freely available specific BIVA software [23].

## 3. Results

Interpopulation differences were observed for all anthropometric and bioelectrical variables, with the Brazilian sample showing greater body dimensions, higher values of Rsp and Zsp, and lower values of phase angle, with respect to both the Mexican, and, especially in the older age class, the Italian ones (Table 1). According to specific BIVA [11] Rsp and Zsp are positively related to FM%, while phase angle is related to ICW/ECW and skeletal muscle mass.

**Figure 1:**
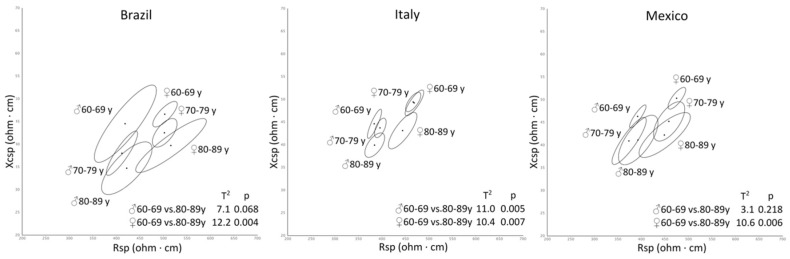
Confidence ellipses for age, sex, and population groups.

In all the three populations, a clear pattern of sex differences and age-related variations was detected (Table 1, Figure 1). Men were taller and heavier than women but had a similar BMI. They also had higher phase angle values and lower values of Rsp, Xcsp, and Zsp.

The oldest individuals showed lower anthropometric, Xcsp, and phase angle values with respect to the youngest ones, while Rsp and Zsp did not change significantly with age.

For both anthropometric and bioelectrical variables, there were no interactions between sex and age, nor between sex and population, or among sex, age, and population, indicating that the pattern of sex differences was similar in the three population groups and that the effect of the aging process was similar in the two genders. The effect of aging was also mostly similar in the three population groups (Table 1, Figure 1). However, a significant interaction was detected for height, that showed a stronger age-related variation in the Italian sample, and for Xcsp and phase angle, that showed a stronger variation in the Brazilian sample.

## 4. Discussion

### 4.1. Population Differences

The three population groups showed differences in body mass and body composition. The Brazilian sample had greater body dimensions, higher relative content of fat mass (as indicated by the higher values of Rsp and Zsp), and lower values of ICW/ECW ratio and skeletal muscle mass (as indicated by the lower phase angles). Both the Mexican and Italian individuals instead had similar body composition, even the Italian sample was characterized by higher weight and BMI.

To our knowledge, there are few population studies focused on the comparison of body composition in older adults [24], especially in older adults living in different countries [9]. Total and regional body composition differences related to ethnicity have been observed along the overall lifespan, including the elderly [25]. For these reasons, the need for ethnic-specific references in this age group has been claimed [9]. However, the overall pattern of body composition differences is not very clear. As to Mexican older adults, some authors observed higher fat mass and lower fat-free or lean mass with respect to other North American, European, or Asian peers [9,24,25]. On the contrary, an extensive analysis of NHANES data (1999–2004) showed slight differences in FM% and BMI values between Mexican American and White American elderly individuals [26]. A disagreement in literature results can be detected for the Italian elderly too. In fact, the FM% values observed in different samples were higher [27], or lower [28] with respect to all population groups considered by NHANES [26]. In this research, both Mexican and Italian older adults showed mean phase angle values similar to the healthy American ones. It should be noted that the phase angle values of the Italian older age classes, as well as their BMI, were relatively high compared to the other population groups, thus indicating better preservation of their health status, as predictable in a population with higher life expectancy. The literature on body composition in the Brazilian population [29] is internally consistent, showing lower FM% among Brazilians with respect to the NHANES groups [26]. In this research, the Brazilian sample showed higher vector length and lower phase angles than both the Mexican and Italian samples. Interestingly, the phase angle values detected in our sample are similar to those observed by Fortes Ferreira et al. [30] among institutionalized older adults in Brazil, and hence could be indicative of their peculiar characteristics.

### 4.2. Sex Differences

The pattern of sex differences was similar in all age groups and in the three populations, and is consistent with the knowledge of the literature on sexual dimorphism, with women showing higher FM% (higher Rsp and Zsp) and lower ICW/ECW ratio and skeletal muscle mass (lower phase angle) with respect to men [11,12,31].

### 4.3. Aging

Despite the differences between sexes and among populations, the trend of age-related variations was similar in the Brazilian, Italian, and Mexican elderly. Lower height, weight, BMI, and phase angle values characterized the older individuals, whereas the vector length remained rather stable.

The literature consistently shows lower body dimensions in the oldest people [32]. Weight loss, mainly due to the reduction of muscle mass, and height reduction, due to osteoporosis, kyphosis, and compression of the intervertebral discs, commonly parallels the aging process, especially in the oldest ages [33].

The literature on age-related variations of body composition also consistently shows a decline in phase angle, with a download migration of specific vectors, that is generally more accentuated in men [14,34]. Such a trend is related to the physiological decline of aging and is mainly due to the loss of skeletal muscle mass [16,33], is related to an expansion of the extracellular water (ECW) compartment [8], and is associated with functional fitness [35]. Indeed, the variations in phase angle can be observed throughout all the life cycle, where they follow the changes of body composition [34], and in athletes, where they increase after a training period [36,37].

The muscle mass decline in older adults is paralleled by the loss of fat mass [33]. In fact, as in the present research, the literature shows that the relative content of FM stabilizes in the decade 55–65 years [26,27,28]. However, a reduction of FM% has been noticed in oldest-old individuals, especially among women [14,38], a tendency that can be recognized among NHANES groups too [26]. The more accentuated reduction of FM% among women and of skeletal muscle mass among men can give rise to a reduction of sexual dimorphism older adults, especially in the oldest old ones [14,38]. A similar interaction between sex and age is not observable in the results of the present study, even if a reduction of the distance between confidence ellipses can be appreciated in the group of >80 years.

The significant interaction between age and population for height is likely related to the more accentuated trend of height reduction in the Italian sample: near 10 cm between 60–69 and 80–89 years, that is near twice than both Brazilian and Mexican older adults. Such observation could be due to the effects of the secular trend, which overlaps those of aging in a cross-sectional study, such as the present one [39]. Indeed, the Italian sample includes a proportion of Sardinian people, who are on average shorter than their Italian peers [40] and have experienced a particularly strong positive secular trend [41]. As to the interaction for phase angle, it is most probably related to the more significant age-related reduction in the Brazilian group, that suggests a more accentuated trend towards sarcopenia, and could be explained with the already discussed peculiar characteristics of this sample.

The present study is subject to limitations. As frequently happens in the research on human aging, the cross-sectional design of the study involves overlap between aging and cohort differences. Furthermore, although standardized procedures were taken into account during the anthropometric and bioimpedance measurements, they were made by different health personnel, so the possibility of inter-observer bias needs to be considered when interpreting the results. Instead, the bias due to the different bioimpedance device in the Brazil cohorts has been adjusted by applying a correction factor. However, it should be stressed that a systematic difference among populations would not have influenced the main result of this study, which is a similar sex- and age-related trend within populations.

As a point of strength, we must emphasize that this is one of the few comparative population studies focused on a large sample of older adults from two different continents.

## 5. Conclusions

This research on older adults living in different countries contributed to a better definition of the complex scenario of population, sex, and age-related differences in body composition variation. Despite their body composition peculiarities, environmental, and demographic differences, the samples of Brazilian, Italian, and Mexican older adults showed a similar pattern of sex differences, with women characterized by a higher relative quantity of fat mass and lower stature, weight, and skeletal muscle mass than men. The trend of age-related differences was also similar among populations and consistent with the literature, with both sexes showing a tendency toward lower skeletal muscle mass with aging. Such a clear trend of age-related variations may contribute to the reduction of sexual dimorphism in the elderly. It is also related to the increased risk of sarcopenia and related health problems.

Because of the practical advantages, specific BIVA can be recommended in the clinical practice for screening and monitoring body composition changes in the elderly.

## Figures and Tables

**Table 1:** Descriptive statistics and two-way ANOVA results for the comparison among sex, age, population groups.

Population	Sex	Age	Height (cm)	Weight (kg)	BMI (kg/m^2^)	Rsp (ohm·cm)	Xcsp (ohm·cm)	Zsp (ohm·cm)	Phase (degree)
Brazilian sample	Men mean (s.d.)	60–69 y *n* = 10	169.5 (5.9)	81.4 (17.0)	28.3 (5.7)	415.5 (65.0)	48.1 (9.2)	418.3 (65.5)	6.6 (0.6)
		70–79 y *n* = 23	166.4 (5.9)	74.1 (11.0)	26.7 (3.4)	408.4 (58.8)	41.4 (9.1)	410.5 (59.3)	5.7 (0.7)
		>80 y *n* = 6	167.4 (2.5)	75.0 (14.9)	26.8 (5.4)	418.7 (75.2)	38.1 (8.4)	420.5 (75.6)	5.2 (0.6)
	Women mean (s.d.)	60–69 y *n* = 68	155.9 (6.4)	69.3 (14.6)	28.6 (5.7)	499.7 (84.5)	50.2 (9.8)	502.3 (84.9)	5.7 (0.6)
		70–79 y *n* = 53	154.0 (5.5)	66.6 (13.4)	28.0 (3.4)	499.2 (74.5)	46.0 (9.5)	501.4 (74.8)	5.3 (0.8)
		80–89 y *n* = 16	150.7 (7.4)	63.7 (12.4)	28.1 (5.4)	512.4 (105.4)	43.5 (9.2)	514.3 (105.7)	4.9 (0.4)
Italian sample	Men mean (s.d.)	60–69 y *n* = 62	167.1 (6.9)	76.3 (11.4)	27.3 (3.4)	384.2 (46.9)	44.6 (9.8)	386.8 (46.4)	6.6 (1.0)
		70–79 y *n* = 137	162.4 (8.0)	70.2 (9.9)	26.6 (3.3)	396.5 (58.0)	43.7 (10.1)	399.0 (58.3)	6.3 (1.2)
		80–89 y *n* = 65	158.0 (7.4)	63.9 (9.1)	25.6 (2.9)	384.7 (67.1)	39.9 (8.6)	386.9 (67.3)	6.0 (1.0)
	Women mean (s.d.)	60–69 y *n* = 75	154.9 (6.6)	64.4 (10.4)	26.8 (3.6)	469.1 (68.3)	49.2 (9.9)	471.7 (68.8)	6.0 (0.7)
		70–79 y *n* = 166	150.9 (6.2)	61.5 (9.7)	27.0 (3.9)	466.5 (83.9)	49.4 (11.1)	469.2 (84.3)	6.1 (1.0)
		80–89 y *n* = 49	144.8 (6.6)	54.9 (10.5)	26.2 (4.8)	444.0 (83.0)	43.1 (11.0)	446.1 (83.4)	5.5 (0.9)
Mexican sample	Men mean (s.d.)	60–69 y *n* = 119	164.4 (6.5)	72.1 (9.5)	26.7 (3.1)	392.2 (77.0)	46.3 (11.1)	395.0 (77.6)	6.8 (0.8)
		70–79 y *n* = 36	163.3 (6.7)	67.7 (9.4)	25.3 (2.9)	373.8 (84.1)	40.9 (12.0)	376.1 (84.7)	6.2 (0.9)
		80–89 y *n* = 11	159.2 (6.3)	61.7 (6.9)	24.4 (2.6)	393.1 (68.6)	41.1 (8.9)	395.3 (68.9)	5.8 (1.0)
	Women mean (s.d.)	60–69 y *n* = 142	153.9 (5.9)	63.1 (8.6)	26.6 (3.4)	475.2 (90.2)	50.3 (11.5)	477.9 (90.7)	6.0 (0.9)
		70–79 y *n* = 42	150.3 (6.1)	58.7 (8.0)	26.0 (3.2)	458.8 (83.6)	45.2 (10.9)	461.1 (84.0)	5.6 (1.0)
		80–89 y *n* = 23	149.3 (6.5)	57.0 (8.6)	25.5 (3.3)	449.3 (97.3)	42.2 (8.8)	451.3 (97.5)	5.3 (0.6)
Inter-subjects effects			*p*	*p*	*p*	*p*	*p*	*p*	*p*
Sex			0.000	0.000	0.102	0.000	0.000	0.000	0.000
Age			0.000	0.000	0.012	0.701	0.000	0.665	0.000
Population			0.000	0.000	0.000	0.000	0.767	0.000	0.000
Age·Sex			0.783	0.312	0.328	0.747	0.698	0.751	0.129
Sex·Population			0.200	0.425	0.573	0.595	0.766	0.601	0.523
Age·Population			0.005	0.392	0.663	0.330	0.045	0.323	0.025
Age·Sex·Population			0.384	0.758	1.000	0.912	0.947	0.913	0.902
